# Nature of Excitons in Bidimensional WSe_2_ by Hybrid Density Functional Theory Calculations

**DOI:** 10.3390/nano8070481

**Published:** 2018-06-29

**Authors:** Hongsheng Liu, Paolo Lazzaroni, Cristiana Di Valentin

**Affiliations:** Dipartimento di Scienza dei Materiali, Università di Milano Bicocca, via R. Cozzi 55, 20125 Milano, Italy; hongsheng.liu@unimib.it (H.L.); p.lazzaroni1@campus.unimib.it (P.L.)

**Keywords:** exciton, self-trapping, photoluminescence, excitonic binding energy, modelling, HSE, transition metal dichalcogenides

## Abstract

2D tungsten diselenide (2D-WSe_2_) is one of the most successful bidimensional materials for optoelectronic and photonic applications, thanks to its strong photoluminescence properties and to a characteristic large exciton binding energy. Although these optical properties are widely recognized by the scientific community, there is no general understanding of the atomistic details of the excitonic species giving rise to them. In this work, we present a density functional theory investigation of excitons in 2D-WSe_2_, where we compare results obtained by standard generalized gradient approximation (GGA) methods (including spin-orbit coupling) with those by hybrid density functionals. Our study provides information on the size of the self-trapped exciton, the number and type of atoms involved, the structural reorganization, the self-trapping energy, and the photoluminescence energy, whose computed value is in good agreement with experimental measurements in the literature. Moreover, based on the comparative analysis of the self-trapping energy for the exciton with that for isolated charge carriers (unbound electrons and holes), we also suggest a simplified approach for the theoretical estimation of the excitonic binding energy, which can be compared with previous estimates from different approaches or from experimental data.

## 1. Introduction

Transition metal dichalcogenides (TMDCs) have been studied for a long time [1,2]. The structure of the progenitor of this class of materials, i.e., molybdenum disulfide (MoS_2_), was determined in 1923 [3]. However, only after the successful discovery of graphene, which has paved the way for the incredibly fast development of the research field on 2D materials, have the bidimensional forms of TMDCs become the object of intense study and activity, especially thanks to their interesting electronic and optical properties [4,5,6]. Indeed, they present strong photoluminescence (PL) and a large exciton binding energy, which make them ideal materials for optoelectronic and photonic applications [7,8,9,10,11,12]. TMDCs are different from graphene and hexagonal boron nitride (h-BN) which have semi-metallic and insulating characters, respectively, as they are semiconductors with a direct band gap in the visible energy range [4,5]. The electronic structure of TMDCs is far more complex than that of graphene or h-BN because of the metal d state’s interaction with the chalcogen p states. This feature can explain the excellent quantum yield of photoluminescence for the single layers that are, thus, particularly suitable materials for LEDs (light-emitting diodes) [13,14]. Due to the low dielectric screening, there is a large Coulomb interaction between the charge carriers (electron and hole), resulting in an unusually high exciton binding energy [8,9,15,16,17]. Understanding the nature of excitons in two-dimensional (2D) TMDCs is of key importance to make use of their optical and charge transport properties in optoelectronic and photonic applications.

**Figure 1:**
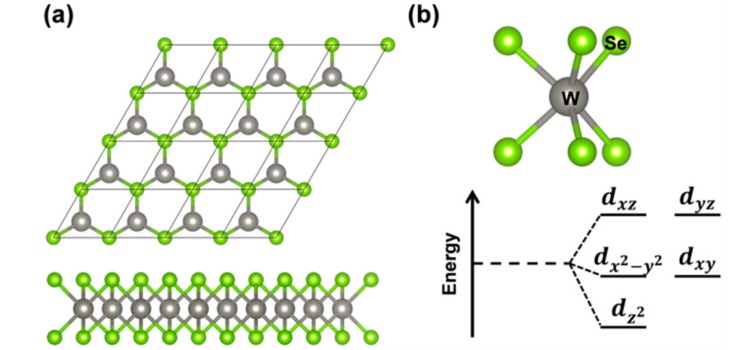
(**a**) Top and side views of a repeated unit cell of 2D tungsten diselenide (2D-WSe_2_); (**b**) A view of the trigonal prismatic coordination and a scheme of the d states splitting in WSe_2_ monolayer deriving from it.

The 2D tungsten diselenide (WSe_2_), which is the object of the present work, belongs to the 2D space group $P\bar{6}m2$ and comprises three atomic layers: The two external layers are triangular sublattices of selenium atoms, whereas the middle layer is a triangular sublattice of tungsten atoms (Figure 1a). The unit cell is hexagonal and is defined by one lattice parameter. Each W atom presents a trigonal prismatic coordination, leading to d states splitting shown in Figure 1b and to the semiconducting nature of this material. Although the electronic structure of 2D-WSe_2_ has been extensively studied from both an experimental and theoretical point of view, there is still some debate on the true nature of the band gap as we will discuss in detail below [18,19]. Calculations were performed at both density functional theory (DFT) and GW levels of theory [5,9,20,21,22]. The main and most peculiar features are related to the presence of two conduction band (CB) minima (at Q and K points in the Brillouin zone) and two valence band (VB) maxima (at K and Γ points in the Brillouin zone). Progressively reducing the number of layers, Zhao et al. observed a band gap inversion from indirect to direct when going from bi- to the mono- (triatomic) layer [13]. Correspondingly, a very large increase in the experimental photoluminescence intensity is registered, which could be assigned to the shift from an indirect to a direct band gap. However, a scanning tunneling spectroscopic (STS) study suggests a minimum indirect band gap (2.20 eV) and the presence of a direct band gap at a very close energy (2.12 eV) [19]. Further complication comes from the sensible difference between optical and electronic gaps [4,5] due to a large exciton binding energy. A typical intense photoluminescence peak deriving from the exciton radiative recombination can be observed at about 1.6–1.7 eV [7,13,23]. In general, when there is a strong coupling between the just formed free exciton and the atomic lattice, self-trapping phenomena takes place, leading to large lattice (polaronic) distortions around the exciton. This atomic relaxation is mainly responsible for the Stokes-shift between the absorption and emission peaks.

The accurate theoretical description of the electronic properties of the ground and excited states of 2D-WSe_2_ is not a simple task. First, local density approximation (LDA) or generalized gradient approximation (GGA) functionals are not well suited to correctly reproducing a semiconductor band gap. One should resort to hybrid density functionals or DFT+U [24,25,26] or GW [27] approaches. The excitation or optical absorption is an even more demanding task. Available theoretical approaches are time-dependent DFT (TD-DFT) [28,29] or the Bethe-Salpeter equation (BSE) [30,31]. The latter includes excitonic effects which are crucial for these types of materials. However, electron-phonon coupling and local relaxation effects cannot be easily included. In this work, we apply a simplified but effective approach that we have used before to model free excitons in semiconducting materials, the exciton trapping process, and finally, the exciton radiative sections. The exciton wave function is computed as the ground triplet recombination [32]. The energy quantities associated with those processes are estimated quite accurately by means of hybrid density functional calculations (HSE06) [33] as will be discussed in the next state of the systems. Then, atomic positions are relaxed to allow for the polaronic distortion. Finally, the spin constraint is released, so that the system can go back to the electronic ground state through a vertical radiative transition. We compare the HSE06 with Perdew-Burke-Ernzerhof (PBE) results, as implemented in the CRYSTAL14 (CRY14) code [34,35]. Moreover, we compare the PBE results based on atomic localized basis sets (CRY14) with those based on plane wave basis sets (Quantum ESPRESSO, QE) [36]. For the latter, we can even introduce the spin-orbit coupling effect, which could be relevant in some respects. To gain a deeper understanding of the physical processes involved, we also investigate self-trapping of separated charge carriers (electron and hole). This will allow, for the first time, a theoretical estimation of the binding energy in a self-trapped exciton in 2D-WSe_2_ to be provided and to be compared to experimentally obtained values in the range between −0.4/−0.8 eV [15,16].

## 2. Computational Details

All the calculations were carried out using the CRYSTAL14 (CRY14) package [34,35] based on density functional theory (DFT) where the Kohn−Sham orbitals are expanded in Gaussian-type orbitals. For selenium and tungsten, we adopted the well-assessed effective core pseudopotential (ECP) techniques [37,38]. The small-core ECP derived by Hay and Wadt [37] was chosen for W, leaving only the 5p, 5d, and 6sp valence electrons to be treated explicitly. For the tungsten valence shell electrons, we used a modified Hay Wadt double-ζ basis set [37], as adopted before for WO_3_ [39]. The ECP and valence shell electrons for Se are treated as reported previously, where the 3s, 3p, 3d, 4s and 4p electrons are viewed as valence electrons [40,41]. Hybrid functional HSE06 [42] was used for both geometry optimization and electronic properties calculations. For the WSe_2_ monolayer, the hexagonal unit cell with the symmetry group of $P\bar{6}m2$ was fully optimized with respect to both atomic coordinates and cell parameters. K point mesh with uniform spacing of 0.015 Å^−1^ was used to get the converged total energy and band gap. The convergence criterion of 0.023 eV/Å for force was used during geometry optimization, and the convergence criterion for total energy was set at 10^−7^ Hartree for all the calculations.

Different sizes of supercell for the monolayer 2D-WSe_2_, including 2 × 2, 4 × 4, 5 × 5, 6 × 6, 8 × 8 supercells, were first tested for the exciton species, which suggested that the 6 × 6 supercell is big enough not to observe spurious effects due to interaction with repeated images. Therefore, we used this throughout the work. To model exciton and charge carriers, triplet and doublet state calculations were performed, respectively. Energy of absorption (Δ*E_ABS_*) is calculated as the energy difference between the triplet and singlet states of the neutral system in its ground state geometry. Self-trapping energies (Δ*E_ST_*) are calculated as the energy difference between the isolated exciton (or charge: e^−^ or h^+^) in the trapping geometry and in the ground state geometry. The vertical emission energy (photoluminescence) (Δ*E_PL_*_)_ is obtained as the difference between the energy of a neutral system in the triplet and singlet states in trapping geometry. The distortion energy (Δ*E_DIST_*_)_ is defined as the difference between the energy of singlet neutral system in trapping geometry and in ground state geometry. Symmetry was removed for these calculations to allow the distortion in geometry induced by exciton or charge trapping.

To compare the results, including spin-orbital coupling, from different basis sets, some calculations are also performed using the plane-wave-based QE package [36]. The projector augmented wave (PAW) potentials [43] were adopted to describe the electron-ion interactions with W (4f, 5s, 5p, 5d, 6s) and Se (3d, 4s, 4p) treated as valence electrons. The exchange and correlation interaction were described by the PBE [44] functional within the GGA. Energy cutoffs of 55 Ryd and 220 Ryd (for kinetic energy and charge density expansion, respectively) were adopted for all calculations. The convergence criterion of 0.026 eV/Å for force was used during geometry optimization, and the convergence criterion for total energy was set at 10^−6^ Ryd for all the calculations.

## 3. Results and Discussion

### 3.1. Atomic and Electronic Structure of 2D-WSe_2_ by Hybrid Density Functional Calculations

We have first assessed the performance of hybrid density functionals in the description of 2D-WSe_2_ since those data are missing in the literature. For this preliminary assessment, we have compared different hybrid functional (HSE06, B3LYP [45,46] and B3PW [45,47]) with PBE results, using the CRY14 code. 2D-WSe_2_ structure belongs to the $P\bar{6}m2$ hexagonal spatial group. The lattice parameters are summarized in Table S1 and are rather close to the experimental value from X-ray diffraction (XRD) of the bulk phase (3.282 Å). In particular, HSE06 outperforms all the others (3.278 Å) with an error of only 0.13%. The correct reproduction of the lattice constant is particularly relevant in the present case since it has been shown that the electronic structure of 2D-WSe_2_ is very sensitive to this parameter. Some artificial compressive/tensile stress leads to an increase/decrease of band gap or to contrasting results on its direct or indirect nature [18].

**Figure 2:**
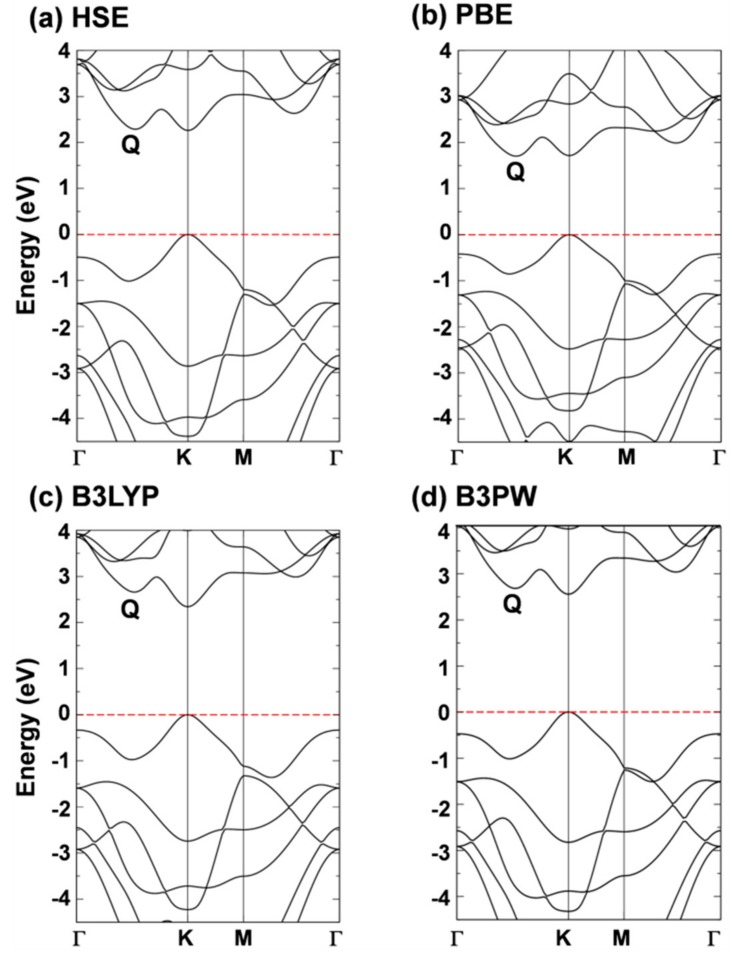
Band structures of monolayer tungsten diselenide (WSe_2_) calculated by (**a**) HSE; (**b**) PBE; (**c**) B3LYP and (**d**) B3PW with CRYSTAL14 code. The Fermi level is scaled to zero (the top of valence band).

Although the W d-states splitting caused by the trigonal prismatic coordination can well explain the basic electronic properties, most of the interesting properties of 2D-TMDCs come from the peculiar dispersion of the band states. In Figure 2, we report the band structure of 2D-WSe_2_ as obtained with the different density functionals. All the hybrid density functionals, in contrast with PBE (K_v_–Q_c_), present a minimum direct (K_v_–K_c_) gap. Moreover, PBE largely underestimated the band gap value (1.71 vs. 2.12 eV [19]) as expected. Although the presence of a direct gap is a hallmark of 2D-TMDCs, for the specific case of 2D-WSe_2_, the crossover between direct/indirect band gap is still under debate. This could be easily rationalized considering the very tiny difference in energy between the two conduction band minima in Q and in K. In HSE and PBE they are practically degenerate since the respective differences of +0.03 and −0.01 eV are within the error of the methods. These differences are very sensitive to the lattice parameter: Note that B3LYP, whose lattice parameter is the largest, provides the largest ΔE (Q_c_–K_c_). From the experimental side, Zhang et al. tried to shed some light on the issue by performing some electronic transport measurements with STS [19]. This work has revealed the existence of both an indirect (K_v_–Q_c_) gap at 2.12 eV and of a direct (K_v_–K_c_) gap at 2.20 eV, with an error range of ~0.1 eV. These data are in good agreement with the HSE06 values as we have just discussed above. HSE06 also provides the best estimate of the ΔE (K_v_–Γ_v_) of 0.49 eV versus the experimental measurement of 0.64 eV [19]. A direct band gap for 2D-WSe_2_ is quite surprising, given the extraordinary PL properties [7]. However, the existence of an almost degenerate indirect band gap can justify the observed phenomenon and is in line with accurate DFT calculations in this work. These results fully agree also with the analysis by Hsu et al. of heterojunction-induced strain effects on PL energy and intensity [18].

**Figure 3:**
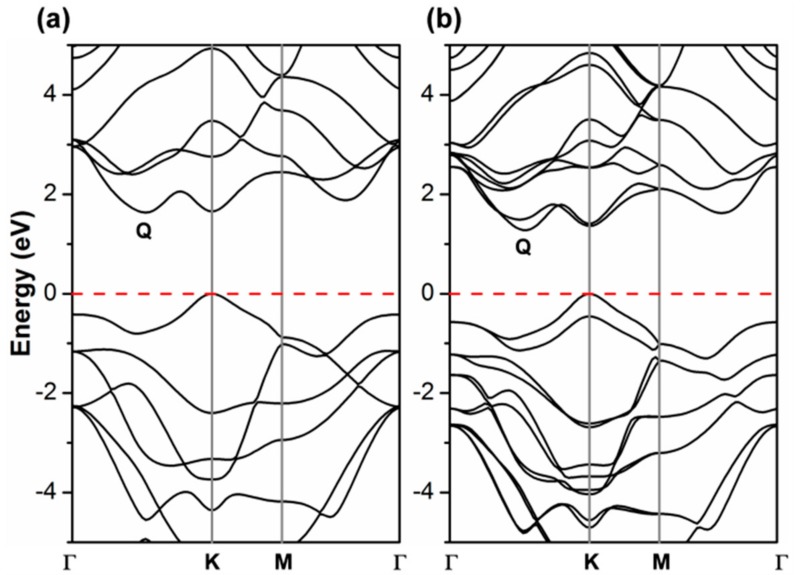
Band structure of monolayer WSe_2_ unit cell obtained by Perdew-Burke-Ernzerhof/Quantum ESPRESSO (PBE/QE) with (**a**) and without (**b**) spin–orbit coupling. The Fermi level is scaled to zero (the top of valence band).

It is noteworthy now to compare the PBE band structure by CRY14 to the corresponding one by QE (Figure 2 vs. Figure 3a). They are essentially identical, which suggests that there is no basis set effect on the band structure. More importantly, it is then relevant to analyze what happens upon the introduction of spin–orbit coupling (SOC) in the PBE/QE calculations (Figure 3b) which cannot be done for CRY14 calculations. We clearly observe a splitting of the α and β states, especially in the valence band at the K point (by 0.46 eV), but not in Γ. In the conduction band, the largest splitting is at the Q point (0.23 eV) and very feebly in K. Our observations are in perfect agreement with a previous work where they have been largely discussed [13,15,16,31,48,49,50,51,52,53,54,55,56,57].

**Figure 4:**
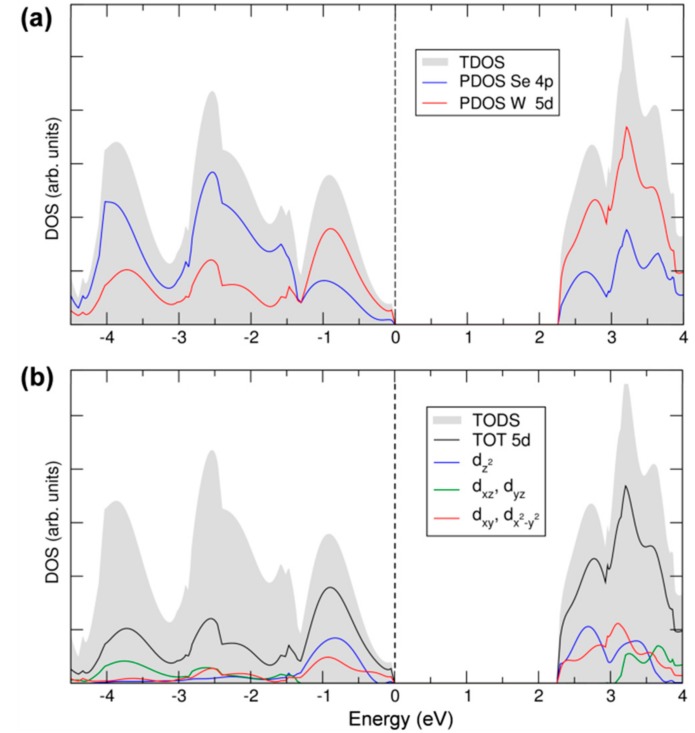
Total and projected density of states (TDOS and PDOS) of monolayer WSe_2_ calculated by hybrid density functional calculations/CRYSTAL14 (HSE06/CRY14). The grey area represents the TDOS. In (**a**), blue and red lines represent PDOS on 4*p* and 5*d* states of Se and W; In (**b**), black, blue, green and red lines represent PDOS on 5*d*, $d_{z^{2}}$, *d_xz_* + *d_yz_* and *d_xy_* + $\;d_{x^{2}-y^{2}}$, respectively. The Fermi level is scaled to zero (the top of valence band).

In the last part of this section, we will discuss the details of 2D-WSe_2_ electronic structure in terms of total and projected density of states (TDOS and PDOS, respectively), which are reported in Figure 4a. The states of the valence and conduction bands are essentially made up by W 5d and Se 4p. The contribution from both elements to both VB and CB suggests a strong covalent character of the W–Se bond in WSe_2_ and, consequently, a strong d-p hybridization. Moreover, we can clearly distinguish a first large peak in the VB, from the Fermi level (set to the zero energy) to −1.5 eV, where the W contribution is predominant, whereas, from −1.5 eV down in energy, the Se contribution becomes the largest, indicating some polarity in the bond, too. The d-p mixing is crucial for the absorption properties of this material since it makes the transition permitted, in contrast to what would happen if top of the VB and bottom of the CB had both a d character since d-d transition are forbidden for the electric dipole selection rules. In Figure 4b, we report the projected density of states on the different *d* states of the W atoms following the trigonal prismatic d splitting. The *d_xz_*_,*yz*_ contribution is negligible in the energy range of the VB close to the Fermi level, where the *d_z_*_2_ and the *d_xy__,x_*_2−*y*2_ are more relevant, in agreement with the crystal field splitting described in Figure 1. At a deeper level of analysis, we can even provide the main contribution of the d states to the highest occupied band and to the lowest unoccupied band, in particular, where at the maximum and minimum points, respectively. The data are presented in Table 1. We can clearly observe that the bands in K_c_ and K_v_ are primarily made up of the W *d_z_*_2_ and the *d_xy__,x_*_2*−y*2_ states. A small contribution comes from the Se *p_x_* and *p_y_* states but not from *p_z_* ones. On the contrary, *p_z_* states contribute to the states at the top of the valence and at the bottom of the conduction band at the Q_c_ and Γ_v_ points, causing the different gap character (direct) for the monolayer system with respect to the bulk. 

### 3.2. Self-Trapped Charge Carriers in WSe_2_

#### 3.2.1. Self-Trapped Electron

**Figure 5:**
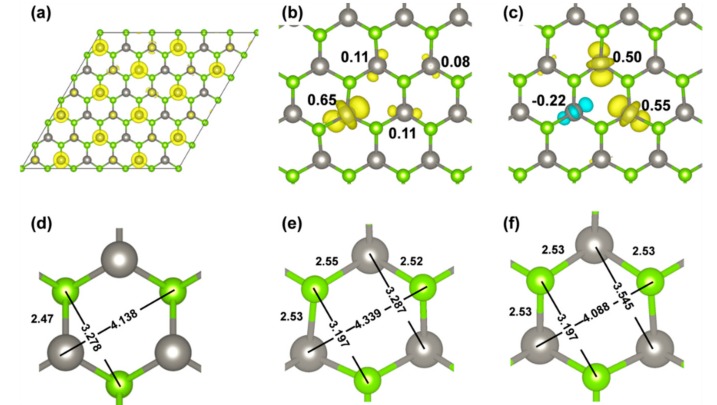
(**a**,**d**) are spin density and geometry of WSe_2_ 6 × 6 supercell with one more electron before geometry relaxation by HSE/CRY14; (**b**,**c**) are spin densities of two solutions for WSe_2_ 6 × 6 supercell with a self-trapped electron; (**e**,**f**) are the local geometries of solution (**b**) and (**c**), respectively. Distances are in Å. The isosurface level is 0.006 electron/bohr^3^ for the spin density plots. Yellow and cyan are positive and negative spin densities, respectively. Mulliken electron spin density values on W atoms are given in (**b**) and (**c**).

An excess electron was added to the neutral and stoichiometric monolayer (here for the 6 × 6 supercell model). It was injected into the conduction band. It was found to be non-uniformly delocalized on several W atoms, as can be clearly observed in Figure 5a. However, when using a hybrid functional (HSE06/CRY14) and upon atomic relaxation, the electron undergoes a self-trapping process with an energy gain of −0.20 eV ($\Delta E_{ST,e^{}}$), as shown below:

$$\;WSe_{2}+1e^{-}\to {\left[WSe_{2}\right]}^{-}\overset{Relaxation}{\to }{\left[WSe_{2}\right]}_{trap}^{-}$$

$$\;\Delta E_{ST,e^{-}}=E_{{\left[WSe_{2}\right]}_{trap}^{-}}-E_{{\left[WSe_{2}\right]}^{-}}$$

During the relaxation, some symmetry is lost, even though we can still observe a perpendicular plane of symmetry, which is represented by a dotted line in Figure 5b. The electron is then found to be localized on three W atoms of one hexagonal ring of the monolayer (65% on one W and 11% on each of the other two, summing to 87% of the unpaired electron density (Figure 5b). There is a further 8% on the W atom lying in the plane of symmetry of the system (Figure 5b). As a point of difference, any attempt to localize the electron by the standard PBE functional was unsuccessful, due to the intrinsic electron self-interaction error, which causes an excessive delocalization. The polaronic distortions leading to the self-trapping with the hybrid functional involve some structural rearrangement reported in Figure 5e to be compared with Figure 5d. We observe an elongation of the W–Se bonds, for those W that host the extra electron, an elongation of the distance between the W with 65% of the extra electron and the Se atom on the opposite side of the hexagon as shown in Figure 5e, and a shortening of the distance between the two Se atoms bound to the W with the 65% of the extra electron.

A second solution for the self-trapped electron exists, which was obtained starting from a different initial atomic configuration and is only 5 meV higher in energy than the one we have just described. For this second solution, some spin polarization is observed, as it is evident in Figure 5c. Again, one plane of symmetry perpendicular to the system layer is kept, however, the electron is mainly localized on the two W atoms at the two sides of the plane (up a component of 50–55%) with some down component (22%) on the third W of the hexagon. The polaronic distortion is similar but with some differences. When we compare Figure 5e with 5f, we observe an elongation of the W–Se bonds again for those W that host the extra electron, an elongation of the distance between the two W atoms with 50–55% of the extra electron, and a shortening of the distance between the W and the Se atoms in the plane of symmetry. 

**Figure 6:**
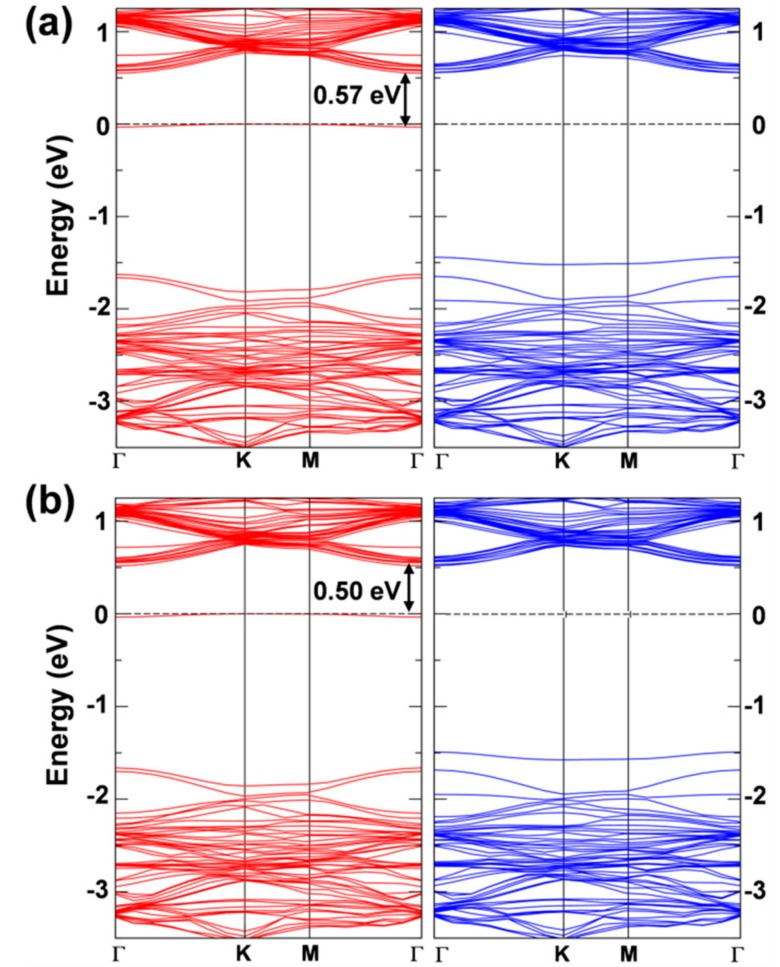
Band structure of the 6 × 6 supercell WSe_2_ monolayer with one self-trapped electron obtained by HSE06/CRY14. (**a**) is for the most stable solution (in Figure 5b) and (**b**) is for the less stable solution (in Figure 5c). Red and blue lines represent the spin up (majority) and spin down (minority) bands, respectively. The Fermi level is scaled to zero. One energy difference at Γ is reported in eV.

The band structure of the two solutions is very similar (Figure 6) and presents a clear flat state in the gap of the spin up component that is occupied by the extra electron. This state features a strong W d character (see Figures S1 and S2 in the Supplementary Literature) and is −0.57 eV below the bottom of the conduction band for the first solution and −0.50 eV for the second one.

#### 3.2.2. Self-Trapped Hole

The removal of one electron from the stoichiometric neutral systems leads to the formation of an electron hole, as shown below:

$$\;WSe_{2}\to {\left[WSe_{2}\right]}^{+}+1e^{-}\overset{Relaxation}{\to }{\left[WSe_{2}\right]}_{trap}^{+}$$

$$\;\Delta E_{ST,h^{+}}=E_{{\left[WSe_{2}\right]}_{trap}^{+}}-E_{{\left[WSe_{2}\right]}^{+}}$$

**Figure 7:**
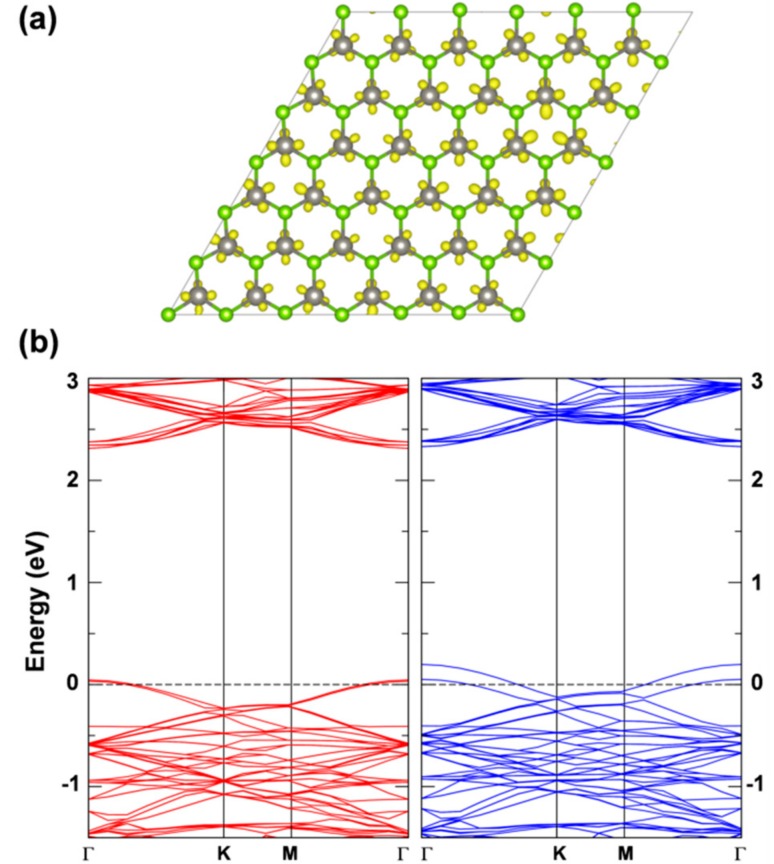
(**a**) Spin density and band structure of the 6 × 6 supercell WSe_2_ monolayer with one hole obtained by HSE06/CRY14. In (**a**), the isosurface level is 0.006 electron/bohr^3^; In (**b**), red and blue lines represent the spin up (majority) and spin down (minority) bands. The Fermi level is scaled to zero.

Any attempt to localize this hole, with supercells models of different size and with both the standard PBE and the hybrid HSE functionals, was unsuccessful (see Figure 7a for the 6 × 6 supercell and HSE). The spin density is found to be fully delocalized on all the W atoms of the model. The relaxed geometry is almost identical to that of the neutral system and the self-trapping energy ($\Delta E_{ST,e^{}})$ is only −0.05 eV. The band structure (Figure 7b) confirms delocalized states in the spin down component crossing the Fermi level. 

### 3.3. Self-Trapped Exciton in WSe_2_

**Figure 8:**
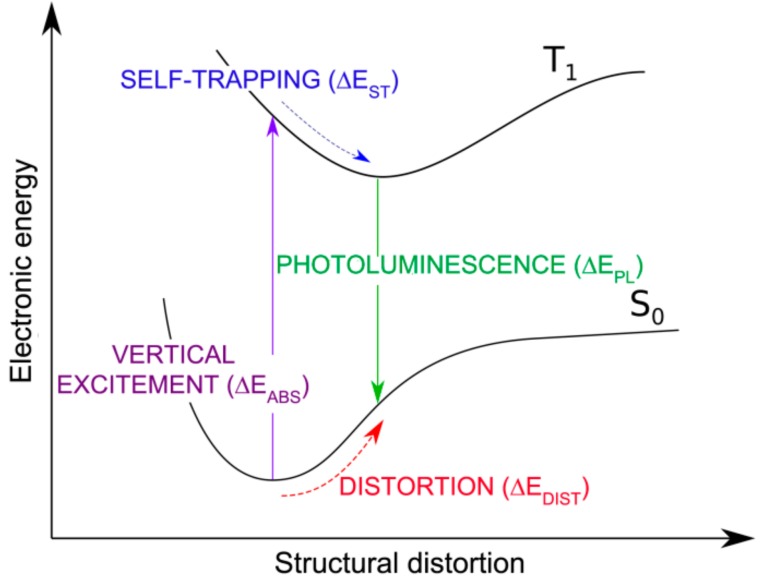
Schematic representation of the S_0_-T_1_ excitation (Δ*E_ABS_*), exciton self-trapping (Δ*E_ST_*) and emission or photoluminescence (Δ*E_PL_*) energies for 2D-WSe_2_.

We now consider the formation, self-trapping and recombination processes of an exciton in a monolayer of 2D-WSe_2_. We define the energy quantities involved in those processes in the scheme of Figure 8. 

We will first present the results obtained with the hybrid functional HSE with CRY14 code on the 6 × 6 supercell model. Density functional theory is a ground state theory. However, we can bypass this critical issue by computing the first triplet state. This is a ground state for the triplet spin configuration and can, therefore, be correctly described by DFT [32]. Therefore, the vertical excitation will be simulated as a S_0_→T_1_ transition ($\Delta E_{abs}=2.44\;\mathrm{eV}$). Although, this is forbidden, it highly resembles the S_0_→S_1_ transition, since, in both cases, one electron has been promoted to the conduction band, where it is largely delocalized and, therefore, its spin character (up or down) is not expected to influence the energy of the system very much when in the same S_0_ ground state atomic configuration. We do not expect that this approximated absorption energy reproduces the experimental value. However, we can use this to obtain the exciton self-trapping energy, as the difference between the energy of the T_1_ state in the S_0_ atomic configuration and the energy of the T_1_ state in its minimum atomic configuration:

$$\;\Delta E_{ST,\mathrm{exciton}}=E_{T_{1}\left(S_{0}\right)}-E_{T_{1}\left(T_{1}\right)}=\;-0.74\;\mathrm{eV}$$

**Figure 9:**
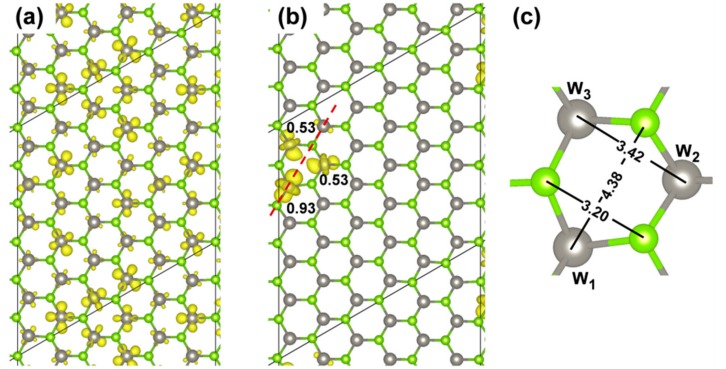
Spin density plots for the triplet state (**a**) before and (**b**) after geometry relaxation calculated by HSE06/CRY14 with a 6 × 6 supercell of WSe_2_; (**c**) Geometry parameters (in Å) after the relaxation due to the exciton. The isosurface level is 0.0015 and 0.006 electron/bohr^3^ for (**a**) and (**b**), respectively. Mulliken electron spin density values on W atoms are given in (**b**).

In the ground state atomic configuration, the T_1_ state is confirmed to be fully delocalized as shown by the spin plot in Figure 9a. Upon geometry optimization, however, the exciton is found to almost fully localize on three W atoms of one hexagon of the system. It is noteworthy to mention that, although we have removed all symmetry constraints, the structure maintains some symmetry, in particular, it keeps a perpendicular plane of symmetry going through a W−Se bond (see dotted line in Figure 9b). 

**Figure 10:**
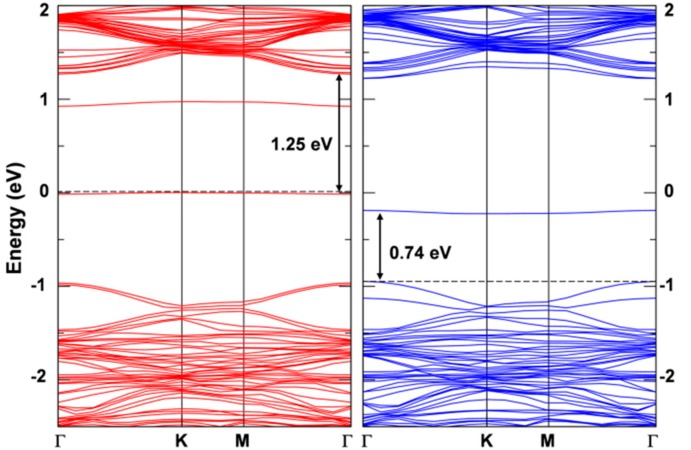
Band structure for the triplet state calculated by HSE06/CRY14 with a 6 × 6 supercell of WSe_2_. The red and blue lines represent the spin up and spin down bands, respectively. The black dashed line indicates the Fermi level and the Fermi level for spin up states is scaled to zero. Some energy difference is reported in eV.

The presence of an exciton leads to two unpaired electrons, one excited in the conduction and one left in the valence band (see the band structure of the T_1_ states in its minimum energy configuration as reported in Figure 10 with the highest occupied band state in the spin up component in red in the middle of the band gap, 1.25 eV below the bottom of the conduction band, and the lowest unoccupied band state in the spin down component in blue about 0.74 eV above the top of the valence band). Therefore, the total spin density must sum to 2. Indeed, we observe a spin localization of 0.93 on one W atom and of 0.53 on the other two W atoms in one hexagonal ring of the lattice (see Figure 9b). We have made several attempts to remove even this residual symmetry operation; however, the system would always go back to this optimized structure, which we consider the atomic configuration of minimum energy for the T_1_ state or triplet self-trapped exciton. 

**Figure 11:**
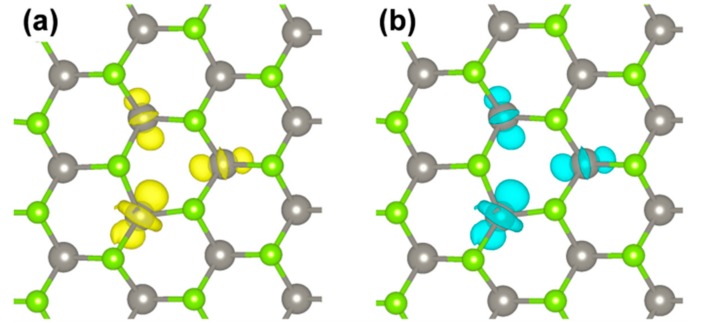
Charge plots for the e^−^ (**a**) and the h^+^ (**b**) in the triplet exciton by HSE/CRY14. The isosurface level is 0.006 electron/bohr^3^.

The spin density plot describes the overall exciton species. However, one would like to distinguish the contribution from the electron and from the hole. This can be obtained following the procedure proposed in Reference [58] by Van Ginhove et al., where the contribution of e^−^ and h^+^ are separated by subtracting the spin up and spin down density of the S_0_ solution from the T_1_ one when they are in the same atomic configuration. The resulting plots for the e^−^ and the h^+^ in the triplet exciton are shown in Figure 11. They actually look quite similar. A fully analogous result is obtained by plotting the highest occupied spin up state and the lowest unoccupied beta state (from the band structure in Figure 10) which are the corresponding one-electron states for the e^−^ and the h^+^ in the triplet exciton.

For the 6 × 6 supercell model, the distance between two repeating excitons is of ~16 Å. We have shown that both the electron and hole are localized on few atoms (three W atoms), which means that the hole is very much attracted by the electron and gets highly localized, in contrast with what was observed for an excess isolated hole in the previous Section 3.2.2. The lattice distortions around the relaxed excitons (Figure 9c) involve both the W−Se bond distance, which are all systematically enlarged, and the W---W or Se---Se or W---Se distances in the hexagon. The hexagon is deformed so that the W---Se distance on the opposite sides becomes 4.38 vs. 4.14 Å in the ground state S_0_ structure (Figure 5d). While the Se---Se distance is reduced to 3.20 Å, the W---W one is enlarged to 3.42 Å, both with respect to 3.28 Å in the ground state S_0_ structure.

Using the data discussed above in this and previous sections, we may now provide a theoretical estimation of the exciton binding energy (*E_B_*), based on electronic structure calculations and density functional theory, according to the following equation:

$$\;E_{B}=\Delta E_{ST,\;exciton}-\left(\Delta E_{ST,e^{-}}+\Delta E_{ST,\;h^{+}}\right)$$

The exciton binding energy is an extremely delicate quantity, which is very difficult to measure and to theoretically compute. From a theoretical point of view, one should resort to the solution of the Bethe-Salpeter equation, but still with the limitation that this would not allow for atomic relaxation and electron-phonon coupling phenomena. On the contrary, here we compute the self-trapping energy of an extra electron and an extra hole in the system as two separated and independent objects, and then we compute the self-trapping energy of the trapped exciton, where electron and hole interact and are bound. This provides a simple route to the exciton binding energy. We obtain a value of *E_B_* = −0.49 eV in excellent agreement with previous experimental and theoretical evaluations in the range between −0.4/−0.8 eV. [9,15,16,17] The large exciton binding energy in this bidimensional material is well explained by the low dielectric screening.

We now suppose that the two charge carriers recombine through a radiative emission. We may compute the photoluminescence energy ($\Delta E_{PL}$) as the vertical transition from the T_1_ state to the S_0_ state in the T_1_ minimum energy configuration:

$$\Delta E_{PL}=E_{S_{0}\left(T_{1}\right)}-E_{T_{1}\left(T_{1}\right)}=\;-1.04\;\mathrm{eV}$$

This energy quantity can more easily be compared to an experimental measurement of 1.6–1.7 eV [7,13,23]. Given the approximations used in this single-particle approach, purely based on DFT total energy calculations, with no electron–phonon coupling treatment, the agreement is satisfactory.

We now wish to comment on the use of supercells of different size and then of different functionals with respect to the exciton description. We have performed the same series of calculations for supercells models of different size (*N* × *N*) going from a 2 × 2, which was proven to be too small for describing the exciton trapping phenomenon, to 4 × 4, 5 × 5, 6 × 6 and 8 × 8. The results are summarized in Table 2 and indicate that they are rather independent on the supercell size, with only minor variations. Not only are the energy parameters similar but also, the spin density values on the relevant trapping W atoms (in Table 2) and the structural distortions (see Figure S3 in the Supplementary Materials). We tested one model with N = odd number, to verify that a different periodicity would not lead to different exciton features, but that was proven not to be the case.

We will now comment on the opportunity of using a different hybrid functional (B3LYP) and then of using a standard GGA functional (PBE). The hybrid HSE06 functional contains 25% of exact exchange in the exchange functional, and it is a long-range screened functional. On the contrary, B3LYP contains only 20% of exact exchange, and it is not screened. Therefore, it is relevant to compare the results of B3LYP with those of HSE to highlight any difference between the two approaches. We observe that B3LYP tend to localize the electron density on the trapping W atoms (0.94, 0.54, 0.54) slightly more with respect to HSE (0.93, 0.53, 0.53). However, the differences are very tiny. Similar considerations can be made on the structural distortions that are reported for B3LYP in Figure S4 in the Supplementary Materials and on the energetics of absorption, self-trapping and photoluminescence that are reported in Table 3. 

**Figure 12:**
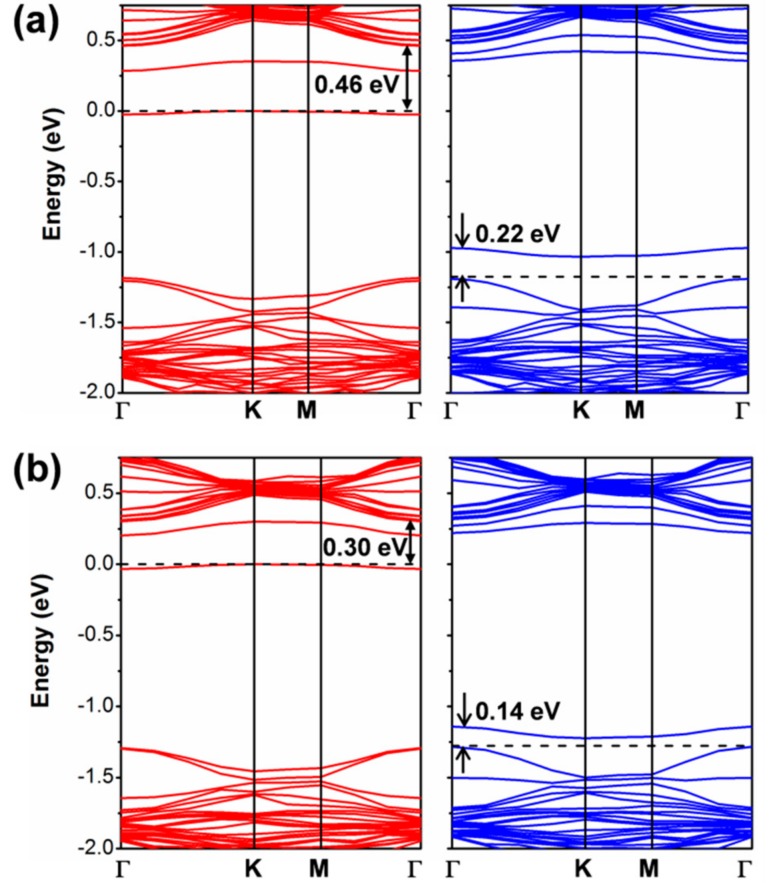
The band structures for the relaxed triplet exciton from PBE calculation by CRY14 (**a**) and QE (**b**). The red and blue lines represent the spin up and spin down bands, respectively. The black dashed line indicates the Fermi level and the Fermi level for spin up states is scaled to zero. Some energy difference is reported in eV.

If we now look at the results with a standard GGA functional such as PBE, the conclusions are rather different. In this case, we tested both an atomic localized basis set with the CRY14 code and a planewave basis set with the QE code. Results with PBE, independent of the type of basis set are characterized by a lower spin density localization for the trapped exciton with respect to what was observed with the hybrid functionals HSE and B3LYP: (0.70, 0.38, 0.38) with PBE/CRY14 and (0.53, 0.30, 0.27) with PBE/QE. However, one can notice that the structural distortion is rather similar to those with hybrid functionals. Energy changes for the cycle in Figure 8, which are reported in Table 3, are rather underestimated, except for $\Delta E_{PL}$, because of the too small band gap by PBE affecting $\Delta E_{ABS}$, and because of the excessive delocalization reducing the trapping effect ($\Delta E_{ST,exciton}$) and enhancing the $\Delta E_{PL}$. Therefore, we may conclude that even the standard PBE functional, which is affected by a severe electron self-interaction error, may qualitatively correctly describe the exciton in 2D-WSe_2_. Note that in the case of bulk excitons in TiO_2_, PBE was found to be totally unsuccessful in the self-trapping process, in contrast with hybrid functionals [32]. We rationalize the unexpected PBE performance on 2D-WSe_2_ as the consequence of an extremely strong and peculiar exciton binding energy. It is indeed an interesting observation and should be compared with the results for separated charge carriers (electrons and holes) for which any attempt of localization and self-trapping process was totally unsuccessful with PBE. The band structure for the relaxed triplet exciton is shown for comparison with the HSE one (see Figure 12). The defect states are clearly less deep in the band gap than for HSE due to the lower degree of trapping. The plot of those defect states is shown in Figure S5 in the Supplementary Materials, as obtained with the PBE/QE calculations. We also show the corresponding density of states (DOS) for these defects, as obtained with PBE/QE and HSE/CRY14 in Figure S6. Again, we observe that the excitonic levels are deeper in the gap for HSE than for PBE, with the Fermi level above the defect state in the spin up component and the Fermi level below the defect state in the spin down component.

## 4. Conclusions

To summarize, in this work we have presented a density functional theory investigation of excitons in 2D-WSe_2_, where we have compared results obtained by standard GGA methods (including spin-orbit coupling) with those by hybrid density functionals. Our study has provided information on the size of the self-trapped exciton, the number and type of atoms involved, the structural reorganization, the self-trapping energy, and the photoluminescence energy, whose computed value (−1.04 eV) is found to be in good agreement with experimental measurements in the literature (1.6–1.7 eV [7,13,23]). In addition, based on the comparative analysis of the self-trapping energy for the exciton with that for isolated charge carriers (unbound electrons and holes), we have also suggested a simplified approach for the theoretical estimation of the excitonic binding energy (−0.49 eV), which can be compared with previous estimates from different approaches or from experimental data (−0.4/−0.8 eV) [9,15,16,17]. To conclude, through this work we have provided the community working with this interesting and useful bidimensional material with an atomistic model of self-trapped exciton in 2D-WSe_2_, whose reliability and accuracy has been corroborated by comparison of computed relevant quantities, such as photoluminescence and exciton binding energy, with available experimental measurements.

## Figures and Tables

**Table 1:** Contribution of atomic orbitals to the valence band maximum (VBM) and to the conduction band minimum (CBM) states projected on different k-points calculated with HSE06/CRY14.

K Point	Main Contribution	Minor Contribution
K_C_	W-$d_{z^{2}}$	Se-*p_x_*, *p_y_*
K_V_	W-$d_{x^{2}-y^{2}},\;d_{xy}$	Se-*p_x_*, *p_y_*
Q_C_	W-$d_{x^{2}-y^{2}},\;d_{xy}$	W-$d_{z^{2}}$; Se-*p_x_*, *p_y_*, *p_z_*
Γ_V_	W-$d_{z^{2}}$	Se-*p_z_*

**Table 2:** Comparison of the energy parameters and spin density for the intermediates involved in the processes schematically described in Figure 8 for different supercells using the hybrid density functional calculations (HSE06/CRY14). The definition of the energy parameters is given in Section 2 of Computational details.

Supercell	Energy Parameters (eV)				Spin Density
	Δ*E_ABS_*	Δ*E_ST_*	Δ*E_PL_*	Δ*E_DIST_*	
4 × 4	2.47	−0.75	−1.04	0.68	0.922 0.534 0.534
5 × 5	2.38	−0.66	−1.04	0.68	0.930 0.530 0.530
6 × 6	2.44	−0.74	−1.04	0.66	0.934 0.533 0.533
8 × 8	2.35	−0.69	−1.03	0.63	0.935 0.535 0.535

**Table 3:** Comparison of the energy parameters for the intermediates involved in the processes schematically described in Figure 8 as obtained with different functionals and a 6 × 6 supercell. The definition of the energy parameters is given in Section 2 of Computational details.

Functional	Energy Parameters (eV)			
	Δ*E_ABS_*	Δ*E_ST_*	Δ*E_PL_*	Δ*E_DIST_*
HSE06/CRY14	2.44	−0.74	−1.04	0.66
B3LYP/CRY14	2.38	−0.81	−0.90	0.67
PBE/CRY14	1.91	−0.17	−1.17	0.57
PBE/QE	1.71	−0.19	−1.30	0.22

## Supplementary Materials

Supplementary Materials are available online at http://www.mdpi.com/2079-4991/8/7/481/s1. Table reporting computed lattice parameters of WSE_2_ with different density functionals. TDOS and PDOS for the electron doped WSe_2_ monolayer. Electron charge density plot of the states hosting the extra electron in the electron doped WSe_2_ monolayer. Ball-and-stick representation reporting atomic distances of the exciton models as calculated with HSE/CRY14 and B3LYP/CRY14. Exciton density of states (DOS). PBE/QE charge density plots of defect states for the self-trapped exciton model.
