# Supplementary material for: Nature of Excitons in Bidimensional WSe2 by Hybrid Density Functional Theory Calculations
Source: Nanomaterials (Basel). 2018 Jun 29;8(7):481. doi: 10.3390/nano8070481 (PMC6070823; doi:10.3390/nano8070481)
Supplement: Supplementary file 1 [file nanomaterials-08-00481-s001.pdf]

Supplementary Materials

Nature of Excitons in Bidimensional WSe_2 by Hybrid Density Functional Theory Calculations

Hongsheng Liu, Paolo Lazzaroni, Cristiana Di Valentin

Dipartimento di Scienza dei Materiali, Università di Milano Bicocca, via R. Cozzi 55, 20125 Milano Italy; hongsheng.liu@unimib.it (H.L.); p.lazzaroni1@campus.unimib.it (P.L.)

* Correspondence: cristiana.divalentin@unimib.it

Table S1. Lattice parameter of monolayer WSe_2 unit cell obtained by different functionals by CRY14 code.

	HSE06	B3LYP	B3PW	PBE
a (Å)	3.278	3.349	3.294	3.306

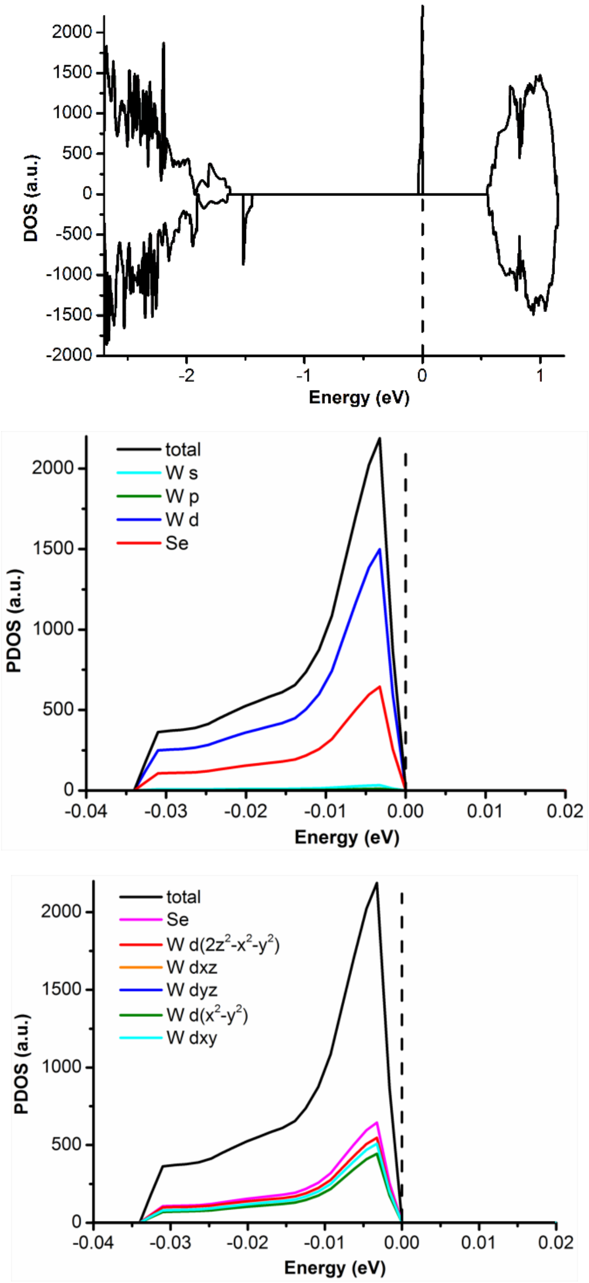

Figure S1. (a) TDOS for the WSE2 monolayer with an extra electron after atomic relaxation as shown in Figure 5b and 5e. (b) PDOS on W s, p, d states and on Se states for the peak at the Fermi level. (c) PDOS on the various W d states and on the Se states for the peak at the Fermi level.

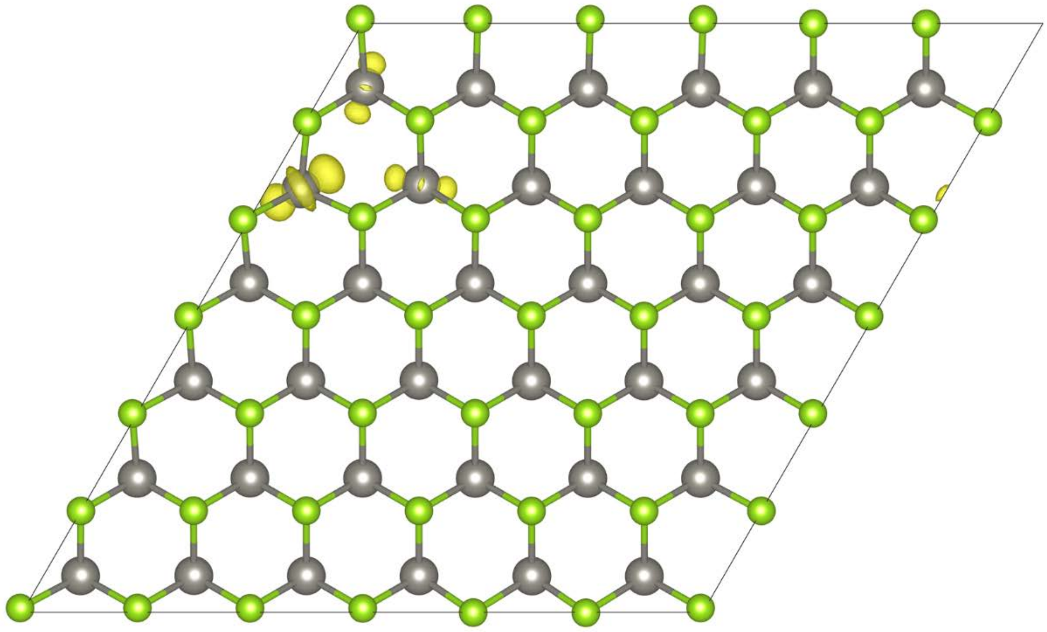

Figure S2. Electron density plot of the highest occupied spin up state shown in the band structure in Figure 6a for an extra electron added to the WSe_2 monolayer in the fully relaxed configuration shown in Figure 5b and 5e. The isosurface level is 0.006 electron/bohr^3.

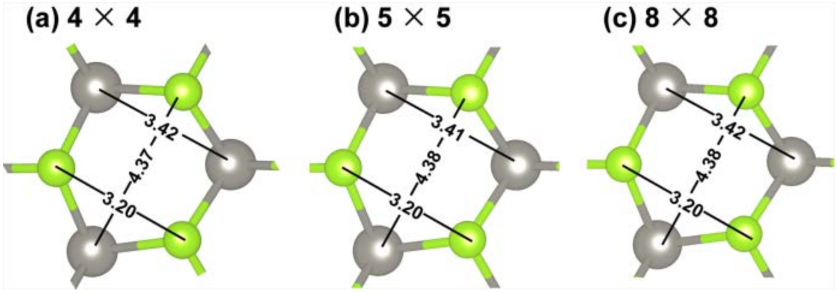

Figure S3. Structural distortions in 4 × 4 (a), 5 × 5 (b) and 8 × 8 (c) supercells of WSe_2 with trapped excitons obtained by HSE/CRY14.

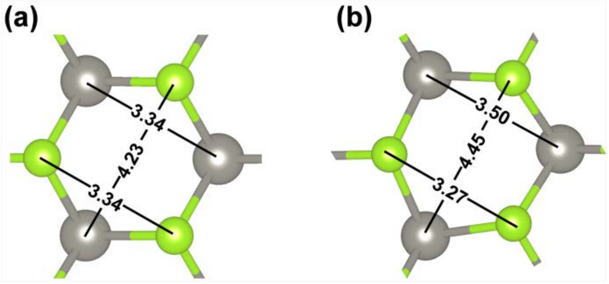

Figure S4. (a) Geometry information for optimized 4 × 4 supercells of WSe_2. (b) The structural distortions in 4 × 4 supercells of WSe_2 with one trapped exciton. The calculations were performed by B3LYP/CRY14.

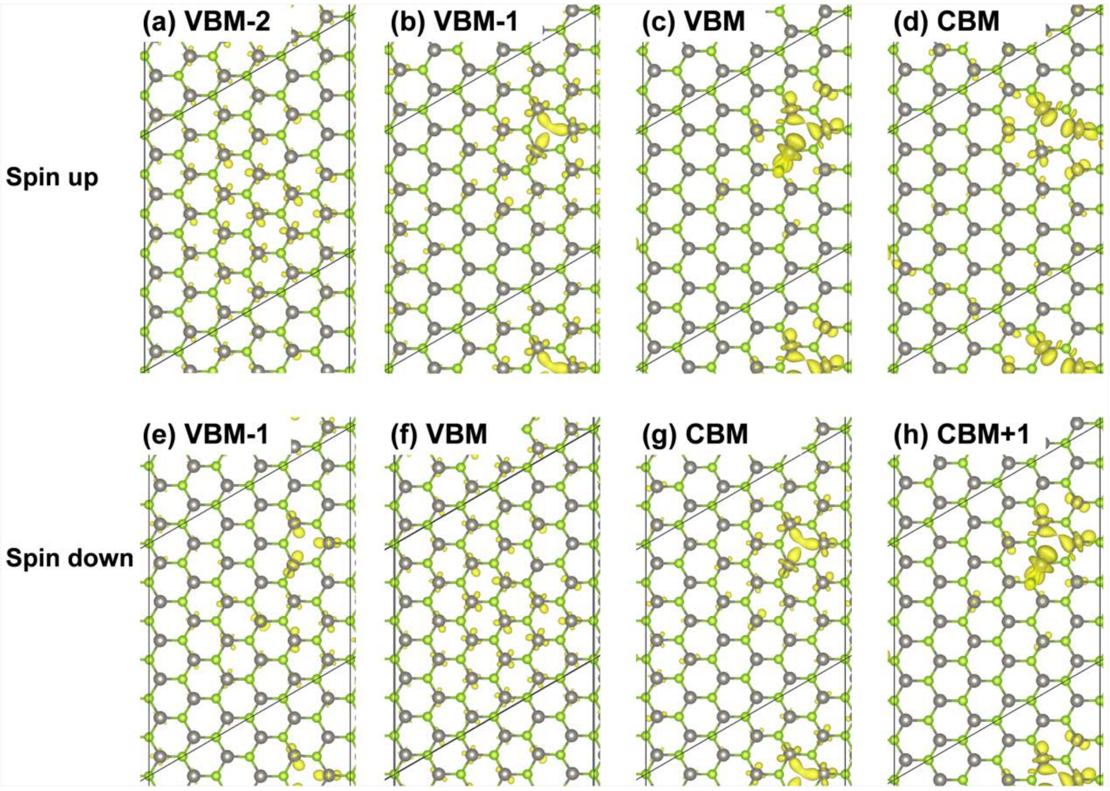

Figure S5. Charge density plots of defect states for the relaxed triplet exciton in WSe_2 6 × 6 supercell calculated by PBE/QE. VBM and CBM are the valence band maximum and conduction band minimum, respectively. VBM±1 refers to one band upper or lower the VBM.

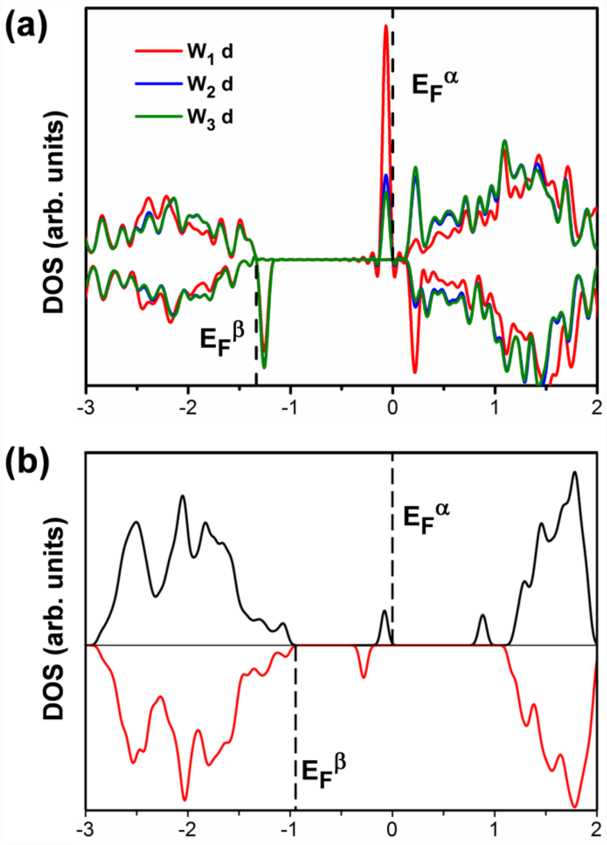

Figure S6. PDOS and TDOS for the triplet self-trapped exciton in 6 × 6 supercell of WSe_2. (a) PDOS on d states of the three W atoms shown in Figure 9c in the manuscript. The calculation was performed by PBE/QE. (b) TDOS obtained with HSE/CRY14. The Fermi level for the spin up state is scaled to zero.
